# Editorial: Telemedicine in ophthalmology: cost-effectiveness and barriers to widespread adoption

**DOI:** 10.3389/fopht.2026.1976103

**Published:** 2026-09-15

**Authors:** Ahmed M. Sayed, Nasif Zaman, Padmaja Kumari Rani

**Affiliations:** 1 Biomedical Engineering Department, Capital University (formerly Helwan University), Helwan, Egypt; 2 Electrical, Computer, and Biomedical Engineering Department, Milwaukee School of Engineering (MSOE) University, Milwaukee, WI, United States; 3 Smith-Kettlewell Eye Research Institute, San Francisco, CA, United States; 4 Anant Bajaj Retina Institute, Srimati Kanuri Santhamma Centre for Vitreoretinal Diseases, LV Prasad Eye Institute, Hyderabad, India

**Keywords:** AI-assisted screening, health care access, refractive errors, screening tools, telemedecine, visual field, workforce

Vision loss remains one of the most preventable yet under-addressed contributors to global disability. A gap exists between diagnostic technology, training and access in modern eye care. Telemedicine is increasingly proposed as the mechanism to close that gap. The five studies gathered in this Research Topic, published between February 2025 and March 2026 and spanning portable perimetry, health-services research, stroke neurology, community health worker training, and artificial intelligence, together offer a useful test of that proposition. Chronologically, they trace an evolving argument: first establishing that remote, low-cost technology can match clinical gold standards, then exposing the structural and human barriers that keep such devices from reaching patients and finally extending the same cost-effectiveness logic to workforce models and AI models.

The sequence opens with the “device” half of the telemedicine equation. Tiang et al. validated a web-based visual field test, Melbourne Rapid Fields (MRF-web), against the Humphrey Field Analyzer in a large sample of patients across metropolitan and rural Australian sites. Concordance was found to be strong (ICC of 0.87 for mean deviation), and diagnostic accuracy for glaucoma was comparable to the standard perimeter, at the cost of roughly one extra minute of test time. Because MRF-web runs on ordinary consumer laptops and is device-independent, it substantially lowers the capital cost of visual field testing and, unlike dedicated perimeters, can plausibly be deployed in a rural clinic or a patient’s home; a direct application of telemedicine principles.

The second article in the topic, authored by Asmare et al., explores the eye care service utilization among older adults. Their community-based analysis from Andabet, Northwest Ethiopia, found that only 16% of older adults had used any eye care service in the prior two years. Multilevel modeling showed that community-level clustering explained most of the variance in utilization, with distance to the nearest facility, health insurance status, and simple awareness of the need for checkups all independently predictive. This accurately resembles the profile of a barrier that telemedicine is meant to address: if the constraint is geographic distance and facility scarcity rather than a lack of willingness to seek care, remote and decentralized service models should help. But the study is also a caution against overstating what connectivity alone can fix, since insurance coverage and basic health literacy were just as decisive as proximity.

The article authored by Wijesundera et al. illustrates a population where the barrier to care is not geography but a systemic blind spot in clinical pathways. Their study used rapid iPad-based bedside testing to show that although 95% of mild-to-moderate stroke patients had normal visual acuity, most had deficits in visual acuity-in-noise, visual fields, or eye-hand coordination, and nearly half were unaware of them. At retest, only about half had fully recovered. Because standard stroke discharge and driving-fitness criteria do not currently include this kind of testing, the study makes an implicit case for portable, low-cost vision test batteries as a routine adjunct to stroke care, a use case telemedicine infrastructure could plausibly support, but one that first requires neurology services to adopt a screening step that does not yet exist in their workflow.

Garba et al. turn to the workforce side of the equation. Their study trained community healthcare workers with no ophthalmic background to operate four portable glaucoma-screening devices in Abuja, Nigeria. After a three-day course, all fifteen trainees passed an 80% competency threshold, with the largest gains in glaucoma knowledge and referral decision-making. This telemedicine-adjacent model proves that it is practical to deploy several portable devices concurrently in primary care settings. Rather than transmitting a live consultation, it pushes screening capacity to the community level, effectively reserving specialist time for referred cases.

Finally, Chen et al., extend the cost-effectiveness argument into software rather than hardware, fine-tuning a multimodal large language model to predict adolescent refractive error from fundus photographs and basic clinical metadata. The model achieved a mean absolute error of 0.647 diopters and generalized well to an independent, temporally separated cohort, and ablation analyses showed it retained reasonable accuracy even without axial length, a measurement that requires dedicated biometry equipment that some screening settings lack. This is a plausible route to lower-cost, asynchronous telescreening for myopia, though the authors themselves flag that the model’s accuracy degraded at extreme refractive ranges and comes from a single, geographically localized center. This study showed that digital health platforms can provide solutions for early myopia screening, making it a potential tool for clinicians practicing in remote areas.

These five studies trace a field maturing in a logical direction: from proving that a low-cost device can match a clinical gold standard, to documenting why access still fails even when good devices exist, to broadening the toolkit with human and algorithmic solutions that might work around those failures. What emerges is that the technical feasibility of low-cost, portable, and AI-assisted ophthalmic screening is now largely established. The harder, unresolved problem remains adoption. Cost savings on hardware do not automatically translate into uptake when insurance coverage is limited, facilities are distant, health literacy is low, or older users struggle with new technologies. The common theme is a shift away from centralized, specialist-dependent models of eye care toward distributed, technology-enabled, and community-embedded ones. Progressing along any of these axes can be helped by first identifying where the standard of care currently lies for each ophthalmic solution. Whether that shift can be sustained, scaled, and reimbursed remains an open question for health systems worldwide, but these five studies collectively make the case that the tools to attempt it already exist.

